# Gender Differences in and the Relationships Between Social Anxiety and Problematic Internet Use: Canonical Analysis

**DOI:** 10.2196/jmir.8947

**Published:** 2018-01-24

**Authors:** Mustafa Baloğlu, Hatice İrem Özteke Kozan, Şahin Kesici

**Affiliations:** ^1^ Department of Special Education Faculty of Education Hacettepe University Ankara Turkey; ^2^ Department of Counseling Ahmet Kelesoglu Faculty of Education Necmettin Erbakan University Konya Turkey

**Keywords:** anxiety, Internet, sex characteristics, social anxiety disorder, addictive behavior

## Abstract

**Background:**

The cognitive-behavioral model of problematic Internet use (PIU) proposes that psychological well-being is associated with specific thoughts and behaviors on the Internet. Hence, there is growing concern that PIU is associated with psychological impairments.

**Objective:**

Given the proposal of gender schema theory and social role theory, men and women are predisposed to experience social anxiety and engage in Internet use differently. Thus, an investigation of gender differences in these areas is warranted. According to the cognitive-behavioral model of PIU, social anxiety is associated with specific cognitions and behaviors on the Internet. Thus, an investigation of the association between social anxiety and PIU is essential. In addition, research that takes into account the multidimensional nature of social anxiety and PIU is lacking. Therefore, this study aimed to explore multivariate gender differences in and the relationships between social anxiety and PIU.

**Methods:**

Participants included 505 college students, of whom 241 (47.7%) were women and 264 (52.3%) were men. Participants’ ages ranged from 18 to 22 years, with a mean age of 20.34 (SD=1.16). The Social Anxiety Scale and Problematic Internet Use Scale were used in data collection. Multivariate analysis of variance (MANOVA) and canonical correlation analysis were used.

**Results:**

Mean differences between men and women were not statistically significant in social anxiety (λ=.02, F3,501=2.47, *P*=.06). In all three PIU dimensions, men scored higher than women, and MANOVA shows that multivariate difference was statistically significant (λ=.94, F3,501=10.69, *P*<.001). Of the canonical correlation functions computed for men, only the first was significant (Rc=.43, λ=.78, χ29=64.7, *P*<.001) and accounted for 19% of the overlapping variance. Similarly, only the first canonical function was significant for women (Rc=.36, λ=.87, χ29=33.9, *P*<.001), which accounted for 13% of the overlapping variance.

**Conclusions:**

On the basis of the findings, we conclude that enhanced educational opportunities for women and their increasing role in the society have led women to become more active and thus closed the gap in social anxiety levels between men and women. We found that men showed more difficulties than women in terms of running away from personal problems (ie, social benefit), used the Internet more excessively, and experienced more interpersonal problems with significant others due to Internet use. We conclude that men are under a greater risk of social impairments due to PIU. Our overall conclusion is that there is a substantial amount of association between social anxiety and PIU and the association is stronger for men than it is for women. We advise that future research continue to investigate PIU and social anxiety as multidimensional constructs.

## Introduction

Adam is a 20-year-old college student who loiters a few minutes in front of his college counselor’s office and rehearses impatiently what he is to say to her. He decides that he will quickly list all his problems once and for all:

I have serious problems and I do not know what to do with them. I’ve found a world on the Internet and I do not let anyone enter into that world. To be honest with you, my mom bugs me to go out more; however, I don't have the guts! I am constantly reminded of the pitfalls of talking to people. The comfort of the Internet is very soothing. I do not know why I am avoiding people. I guess I fear that the more I open up to others, the more I give them to criticize me. I mean everything about me…the way I dress, talk, act…everything! I cannot live with such criticism! Whenever I am being criticized I feel worthless. I am not good at ice breakers, you know. But the Internet is not like that. Nobody really knows who you are on the Internet. I can say anything I wanna and nobody knows who you are! I can post a handsome picture on my profile and have more time to think before I say what I wanna to say. I get more ‘likes’ and feel accepted. I do not think anything is wrong with that! But my mom says I have problems and I've got to see a shrink. What do you think?

While he was rehearsing, the door opens and the counselor invites him in. All he was thinking was suddenly gone out of his mind and he starts sweating and shaking. He was not even able to look her in the eye…

The use of the Internet may result in healthy (ie, positive) or unhealthy (ie, negative) consequences [1]. Internet use can be identified with positive behavioral changes, psychological comfort, and the ease of accessing the available material. On the other hand, problematic Internet use (PIU) may lead to academic, social, occupational, or psychological impairments [2]. The distinction between positive and negative use of the Internet primarily depends on the amount of time spent and the types of activities engaged on it [3].

### Problematic Internet Use

PIU is a comprehensive term that encompasses various unhealthy consequences of the Internet, including negative use, social benefits, excessive use, addiction, social comfort, depression, impulse control, and distraction [4]. Beard and Wolf [5] assert that PIU is a more encompassing construct compared with the term Internet addiction, even though the two share a few common characteristics and may be used interchangeably in the literature. They argue that PIU does not necessarily contain some of the symptoms that are available in Internet addiction. We opt to study PIU in our research.

The cognitive-behavioral model of PIU proposes that psychological well-being is associated with specific cognitions and behaviors on the Internet [4,6,7]. Hence, there is growing concern that PIU is associated with psychological impairments [2,8]. Numerous measurement instruments have been developed to objectively assess PIU (eg, [9,10]). The detrimental effects of PIU on psychological well-being are documented in the literature (for a review see [1]).

According to the displacement hypothesis, time spent on the Internet takes away time spent in real-life interactions [11]. Thus, psychological problems may occur when one engages more in Web-based relationships than real-life interactions [12]. Furthermore, *the lonely drawn to the Internet* hypothesis proposes that individuals who feel lonely and are socially more anxious engage in Web-based activities as a solution [13]. Studies on loneliness, shyness, and social anxiety show that Web-based communication may be sought as a potential escape (eg, [14]). In addition, insecurity is also found to have significant effects in the development of PIU (eg, [15]).

Socially anxious individuals seek environments in which they can engage with others more conveniently. The Internet is an available ground for such people to disclose their inner selves, interact with others, and gratify their needs *.* However, Internet use may become problematic for individuals in different ways. A review of the relevant literature based on gender schema theory and social role theory shows that men and women experience social anxiety and PIU differently. Therefore, this study aimed to report multivariate gender differences in social anxiety and PIU. In addition, we explored the interaction between social anxiety and PIU across men and women.

### Social Anxiety

According to the *Diagnostic and Statistical Manual of Mental Disorders* (*DSM-5*) [16], individuals who have difficulties in forming and maintaining relationships may reveal anxious attitudes in “social relationships.” In this respect, *social anxiety* refers to a strong fear of being judged by others and the corresponding feelings of shame [17]. Social anxiety has been the subject of numerous studies in recent years [18]. Studies usually show that individuals with intense fear of being evaluated by others and those who worry extensively about being criticized in social situations prefer environments in which they can reveal themselves more comfortably [19]. The lonely drawn to the Internet hypothesis proposes that one such medium is the *Internet.* Those who experience difficulties in face-to-face communication prefer Web-based relationships [20]. Therefore, the Internet is an important source in coping with social anxiety, and it provides opportunities for social communication [21]. Additionally, technology-based applications are effectively used in the assessment [22] and treatment [23] of social anxiety.

Studies on social anxiety and Internet use show that individuals facing considerable difficulty in social interactions tend to use the Internet more frequently [15]. Given the proposal of the cognitive-behavioral model of PIU and the existence of significant associations between psychological health and PIU in previous studies, an investigation of the relationships between social anxiety and PIU is warranted.

Gender differences are partially determined by sociocognitive factors [24] such as social roles (ie, [25]) and gender roles (ie, [26,27]). According to gender role theory [28,29], different attributes are attached to femininity and masculinity in the culture.

**Table 1:** A summary of the problematic Internet use literature.

Study	Participants	Analysis	Major finding
Cao et al [43]	17,599 Chinese adolescents	*t* test	Males were higher than females in PIU^a^
Hetzel-Riggin and Pritchard [44]	425 American undergraduates	Hierarchical multiple regression	Social anxiety was a predictor for men’s PIU, but depression was a predictor for women’s PIU
Kormas et al [45]	866 Greek adolescents	Descriptive statistics	Maladaptive Internet users are more likely to be males
Mottram and Fleming [46]	272 Australian undergraduates	Multivariate analysis of variance	Males report more problems related to Internet use than females
Durkee et al [47]	11,956 adolescents in different European countries	Descriptive statistics	Female students were higher in maladaptive Internet use, whereas males were higher in pathological Internet use
Schimmenti et al [48]	310 Italian high school students	Descriptive statistics	Males showed more risky behaviors in PIU

^a^PIU: problematic Internet use.

For example, athletic, competitive, dominant, and aggressive are a few of the attributes of masculinity, whereas affectionate, sensitive, warm, and sympathetic are some of the common attributes of femininity in many cultures. Gender role theory has eventually evolved to gender schema theory [30,31], which purports that individuals develop gender-appropriate cognitive schemas early in childhood through social learning to behave consistent to their biological gender [32]. In addition, social role theory considers gender schemas as dynamic structures of the culture and hypothesizes that individuals display different social roles depending upon their societal and cultural surroundings. For example, we tend to behave quite differently when we are among family members from when we are among colleagues. Social role theory indicates that the expectations of the culture from men and women reveal themselves in the use of power and statute [33] and *gender role beliefs* are shaped by the perceptions of the social expectations [34]. Therefore, according to Bussey and Bandura [24], gender-relevant outcomes are social rather than instinctual. In sum, cognitive gender schemas largely affect individuals’ thought processes and behaviors (eg, [26,29,31]) and eventually gender plays a significant role in explaining differences in social anxiety as well as PIU. Therefore, gender differences in psychological health (eg, [35]) and Internet use (eg, [36]) have long been investigated.

As we summarized above, cognitive and behavioral constructs prime men and women to think, feel, believe, or act differently. Anxiety is one such emotion that men and women experience differently. Spielberger [37] conceptualized anxiety as trait anxiety and state anxiety; in which the former refers to stable individual differences in anxiety proneness, whereas the later refers to a content-dependent, transitory emotional condition. Although studies are not in complete consensus, research largely indicates both in general anxiety [35] and various types of state anxieties, women score higher than men (eg, [38-40]). Social anxiety can also be regarded as a specific type of state anxiety. Even though a few studies found the contrary (eg, [41]), most epidemiological and community-based investigations have found that the prevalence of social anxiety is higher among women [42]. Therefore, we hypothesized that women would score higher on social anxiety than men.

Gender differences have also been studied in relation to the Internet. Studies largely show that men are more likely to use the Internet and encounter higher levels of PIU than women (see Table 1). Therefore, we also hypothesized that men would score higher on PIU than women.

In sum, given the proposal of gender schema theory and social role theory, men and women are predisposed to experience social anxiety and use the Internet differently. Therefore, an investigation of gender differences in these areas is warranted. According to the cognitive-behavioral model of PIU, social anxiety is associated with specific cognitions and behaviors on the Internet. Therefore, an investigation of the association between social anxiety and PIU is essential. In addition, research that takes into account the multidimensional nature of both social anxiety and PIU is lacking. Therefore, we developed and tested the following research hypotheses.

### Hypotheses

Bem [30,31] theorized that individuals develop cognitive schemas throughout childhood to display gender-appropriate behaviors. Such cognitive schemas eventually predispose men and women to reason and behave differently. For example, it is well documented that women place more value on the views of others regarding their own appearances [49]. Thus, it can be hypothesized that women would suffer more from social anxiety. Similarly, researchers show that men and women behave differently on the Internet. Durkee et al [47] found that male adolescents preferred Web-based games, whereas female adolescents preferred social interactions such as chat rooms on the Internet. In addition, Weiser [50] found that men use the Internet more for entertainment purposes, whereas women use it more for interpersonal communication. On the basis of such previous findings, we hypothesized that women would score significantly higher than men in social anxiety components (hypothesis 1), but men would score significantly higher than women in PIU (hypothesis 2).

According to the displacement hypothesis, because socially anxious individuals spend less time with others in real life, they will tend to dwell more on the Internet, leading to more serious consequences of PIU. Supporting these theories, Fehm et al [19] conclude that people who experience negative feelings tend to prefer safer environments such as the Internet. In addition, it can be postulated that the Internet provides a secure environment for individuals who display the symptoms of social anxiety. Similarly, Weinstein et al [18] found statistically significant associations between social anxiety and Internet addiction in two samples of young adults (*r*=.41 and *r*=.34, *P*<.001). Chiang and Hsiao [20] indicated that individuals who prefer Web-based communication tend to also show more PIU. However, only a few studies investigated the relationship between PIU and psychological well-being separately for men and women. Huang [1] advises that the relationship between PIU and psychological well-being should be explored across men and women. Therefore, we hypothesized that there would be a positive relationship between social anxiety dimensions and PIU dimensions both for men and women (hypothesis 3).

## Methods

### Participants

Because social anxiety is hypothesized to manifest from midteen years [16] and because PIU is more prevalent among college students [51], we decided to include a group of college students in our sample. A total of 505 college students who were enrolled in classes in two large state universities in Turkey voluntarily participated in the study. Of the group, 47.7% (241/505) were women and 52.3% (264/505) were men. Participants’ ages ranged from 18 to 22 years, with a mean age of 20.34 years (SD=1.16). None of the participants were clinically diagnosed in terms of either social anxiety or PIU. The number of hours of Internet use among participants ranged from 1 to 105 hours per week with a mean of 14.84 hours (SD 15.94).

### Measures

The Social Anxiety Scale (SAS) and the Problematic Internet Use Scale (PIUS) were used to collect the data. The SAS was developed by Özbay and Palancı [52] to assess social anxiety within the frame of social avoidance, criticism anxiety, and self-depreciation. The scale includes 30 Likert-type items, and higher scores on the total and subscales indicate higher levels of social anxiety, social avoidance, criticism anxiety, and self-depreciation. The psychometric properties of the SAS were found adequate [52]. Internal consistency of the SAS and its subscales were found acceptable in the current sample for both men and women (Table 2).

The PIUS was developed by Ceyhan et al [53] as a 33-item, 5-point Likert-type self-report instrument and includes the following three subscales: negative use, social benefit, and excessive use. Higher scores on the scale indicate higher levels of PIU. The scale was found to have adequate validity and reliability properties [53]. Internal consistency of the PIUS and its subscales was found to be adequate in the current sample for both men and women (Table 2).

### Procedure

Human Subjects Committee had reviewed and approved the study protocol before we started the data collection procedure. Students were recruited in their classes and informed about the purposes of the study. After signing the consent form, the counterbalanced research packets were administered during class hours, within approximately 30 min. Data were screened for the assumptions of parametric statistics. Multivariate outliers, normality, and homogeneity of variances were tested. Gender differences on the subscales of the SAS and the PIUS were investigated using multivariate analysis of variance (MANOVA). In addition, two separate canonical correlation analyses were conducted to investigate how a set of social anxiety variables was related to a set of PIU variables independently for both men and women.

**Table 2:** Descriptive statistics for the Social Anxiety Scale (SAS) and the Problematic Internet Use Scale (PIUS) for men and women.

Variables^a,b^	SAS	Social Avoidance	Criticism Anxiety	Self-Deprecation	PIUS	Excessive Use	Social Benefit	Negative Use
Social Avoidance	.94 (.94)							
Criticism Anxiety	.90 (.93)	.76 (.80)						
Self-Deprecation	.87 (.90)	.72 (.76)	.68 (.78)					
PIUS	.39 (.28)	.37 (.22)	.31 (.24)	.39 (.36)				
Excessive Use	.27 (.14)	.25 (.10)	.24 (.11)	.25 (.21)	.78 (.77)			
Social Benefit	.42 (29)	.38 (.23)	.35 (.25)	.40 (.35)	.89 (.85)	.61 (.51)		
Negative Use	.37 (.28)	.34 (.21)	.27 (.24)	.37 (.35)	.96 (.95)	.66 (.63)	.79 (.71)	
Mean	2.59 (2.50)	2.61 (2.50)	2.79 (2.76)	2.31 (2.18)	2.00 (1.67)	2.64 (2.39)	1.99 (1.64)	1.78 (1.44)
Standard deviation	.66 (.66)	.77 (.80)	.67 (.77)	.74 (.77)	.76 (.61)	.88 (.87)	.78 (.65)	.85 (.65)
Alpha	.92 (.94)	.86 (.89)	.76 (.84)	.75 (.80)	.95 (.94)	.71 (.76)	.85 (.83)	.94 (.93)

^a^*r≥*.15, *P=*.01; .12< *r*<.14, *P=*.05.

^b^within parentheses are values for women.

## Results

### Descriptive Findings

Descriptive statistics for the SAS and the PIUS for men and women were computed and reported in Table 2. Subscale scores were divided by the number of items in the respective subscales to derive subscale means independent of the number of items. Results show that in social anxiety, both men (mean 2.79, SD 0.67) and women (mean 2.76, SD 0.77) scored the highest on the criticism anxiety subscale and both men (mean 2.31, SD 0.74) and women (mean 2.18, SD 0.77) scored the lowest on the self-depreciation subscale. However, mean differences between men and women were not statistically significant (λ=.02, *F*_3,501_=2.47, *P*=.06). Therefore, results failed to support the first hypothesis. On all three PIU dimensions, men scored higher than women (Table 2). MANOVA shows that multivariate difference was statistically significant (λ=.94, *F*_3,501_=10.69, *P*<.001). Thus, these results support the second hypothesis.

### Canonical Findings

Before canonical correlation analyses, multivariate outliers were screened by Mahalanobis distances. Any Mahalanobis distance greater than 22 (χ^2^_6_=22.5, *P*<.001) was considered to be a multivariate outlier. A total of five cases were found to be multivariate outliers and excluded from the further analyses. After multivariate outliers were deleted, Mardia coefficient provided an indication that the data were free from serious violations of multivariate normality (Mardia coefficient=6.57; normalized estimate=7.54). Multivariate homoscedasticity was investigated by Box M statistics, and results show that observed covariance matrices of the SAS and the PIUS were not equal across genders (Box M=45.53, *F*_21,894734_=2.14, *P*=.002).

Two separate canonical correlation analyses were performed, one for men and one for women, to investigate multivariate relationships between social anxiety variables (ie, social avoidance, criticism anxiety, and self-depreciation) and PIU variables (ie, excessive use, social benefit, and negative use). Table 2 presents the Pearson product-moment correlation matrix from which canonical roots were generated. Three canonical functions were computed for men, and three canonical functions were computed for women. The strength of the relationship was assessed by the magnitude of the canonical correlation coefficients. Of the three canonical correlation functions computed for men, only the first was significant (*R*_c_=.43, λ=.78, χ^2^_9_=64.7, *P*<.001) and accounted for 19% of the overlapping variance. Similarly, only the first canonical function was significant for women (*R*_c_=.36, λ=.87, χ^2^_9_=33.9, *P*<.001), which accounted for 13% of the overlapping variance. Therefore, canonical correlation results support the third hypothesis for both men and women. For both groups, standardized canonical coefficients, the percentage of variances accounted in each set, and redundancies were computed and reported in Table 3. The proportion of shared variance together with redundancy indicates that the first canonical variate was moderately related for men. The canonical variate accounted for 64% of the variability in the social anxiety set and 53% in the PIU set. For women, the canonical variate accounted for 41% of the variability in the social anxiety set and 50% in the PIU set.

**Table 3:** Canonical correlation results.

Variables		First canonical variate			
		Men		Women	
**Social anxiety set**		*r*_s_^a^	Coefficient^b^	*r*_s_^a^	Coefficient^b^
	Social avoidance	−.90	−.39	−.69	.06
	Criticism anxiety	−.81	−.11	−.67	.22
	Self-depreciation	−.95	−.60	−.99	−1.20
	Variance percentage	.80		.64	
	Redundancy	.15		.08	
**Problematic Internet use set**					
	Excessive use	−.64	−.02	−.63	.01
	Social benefit	−.98	−.70	−.93	−.53
	Negative use	−.91	−.36	−.94	−.55
	Variance percentage	.73		.71	
	Redundancy	.14		.09	
Canonical correlation coefficient (*R*_c_)		.43		.36	
*R*_c_^2^		19%		13%	

^a^Structure coefficients (canonical loadings).

^b^Standardized canonical coefficients.

A cut-off score of .30 is suggested as minimum acceptable loading value (ie, structure coefficients) in interpreting the variables of a given canonical variate. All structure coefficients in all sets for both men and women exceeded the acceptable cut-off limits (Table 3). For both men and women, the highest canonical loading in the social anxiety set was self-depreciation (−.95 and −.99, respectively) and the lowest was criticism anxiety (−.81 and −.67, respectively). For men, the highest canonical loading in the PIU set was social benefit (−.98), whereas it was negative use (−.94) for women. For both men and women, the lowest PIU was excessive use (−.64 and −.63, respectively). The pair of canonical variates shows that higher self-depreciation, social avoidance, and criticism anxiety are most associated with greater social benefits for men. However, for women, higher self-depreciation, criticism anxiety, and social avoidance are most associated with greater negative use.

## Discussion

### Principal Findings

Descriptive results revealed that even though men scored higher than women in the components of social anxiety, multivariate analyses showed that differences were not significant. Therefore, we rejected the first hypothesis. In general, social anxiety studies show gender differences where women suffer more from social anxiety (eg, [43]); however, given the contemporary era, culture, and the characteristics of the present population, such gender differences have reduced to a point where they are not significant anymore. This is one of the major contributions of this study to the existing literature. Our findings support those studies that did not find any significant gender differences in social anxiety (eg, [36]). One plausible explanation for the nonsignificant gender difference in social anxiety is that contemporary gender roles have become more similar in recent years. Enhanced educational opportunities for women or their increasing role in the society have lead women to become more active and thus closed the gap in social anxiety levels between men and women. However, this conclusion needs further support by future studies.

Significant differences were found in PIU. Men’s averages were higher than those of women’s averages in excessive use, social benefit, and negative use. Even though some studies reveal the contrary or no difference, there is stronger research evidence that men tend to experience higher levels of PIU (Table 1). We aimed to clarify such discrepancies in the literature. Our results continue to support the earlier as well as more recent research findings showing higher PIU among men. We conclude that men are under more risk of PIU. Majority of the current studies in the literature have investigated PIU as a unidimensional construct (eg, [13,48]). However, one of the unique contributions of this research is that it viewed PIU as a multidimensional construct. We found that men showed more difficulties than women in terms of running away from personal problems (ie, social benefit), used the Internet more excessively, and experienced interpersonal problems with significant others due to Internet use. Therefore, we conclude that men are under greater risk of social impairments due to PIU. These results support gender schema theory, which assert that gender affects individuals’ cognitions and behaviors not only in conventional settings but also on the Internet. We can say that our results extend the implications of gender schema theory and social role theory to the virtual domain.

This study is the first in the literature that considered the multivariate nature of both social anxiety and PIU in studying the relationship between the two. We found significant multivariate relationships between sets social anxiety variables and sets of PIU variables for both men and women and thus supported the third hypothesis. These relationships support the cognitive-behavioral model of PIU and indicate that psychological health (ie, social anxiety) and certain problematic behaviors on the Internet are associated to each other. Also supported by the findings is the displacement hypothesis. As the levels of social anxiety increase and people spend less time in real-life relationships and more time on the Internet, risk of developing PIU becomes greater.

This study found specific relationships between certain aspects of social anxiety and certain aspects of PIU for men and women. For example, social anxiety components are mostly related to social benefit problems among men; however, they are most relevant to negative use problems among women. These results support the finding of Hetzel-Riggin and Pritchard [44], which show that men use the Internet more for seeking social support. Finally, results also lend support to the lonely drawn to the Internet hypothesis, indicating that greater social problems may lead to higher Internet use.

The strength of the association between the two constructs can be assessed by squared multiple correlations, which show the amount of shared variability. In this study, 19% and 13% variability were shared between social anxiety and PIU for men and women, respectively. According to guidelines suggested by Cohen [54], these effect sizes can be considered large. The magnitudes of highly significant canonical correlations (averaged *R* of .40) indicate that there is a substantial amount of association between social anxiety and PIU. It can also be concluded that association between social anxiety and PIU is stronger for men than it is for women.

Results indicate that men or women who are more self-depreciative, who avoid social contact, and who suffer from higher levels of criticism anxiety tend to use the Internet more for social benefit and engage in more negative and excessive use. Similar to the findings of this study, Peter et al [55] reported that Internet communication is highly valued by socially anxious adolescents. In sum, we conclude that social anxiety is an important factor in PIU. People with higher levels of social anxiety may fulfill their needs on the Internet and seem to prefer Web-based communication, but the patterns of such a fulfillment vary between men and women.

We also found that both social anxiety and PIU canonical variates were strongly related to all the variables that form their respected variates. The proportions of variances and redundancies indicate that the social anxiety canonical variate was stronger in men than in women. In PIU, canonical variates were almost identical among men and women. For both genders, self-depreciation was the highest loading variable in social anxiety. This finding can be interpreted to mean that being unhappy with oneself contributes the most to social anxiety. However, in PIU, social benefit contributes the most to social anxiety for men, but for women, negative use contributes the most to social anxiety. Additionally, these significant relationships were invariant across men and women even though the association was stronger for men than it was for women.

### Implications

A number of implications from the findings of the study are to be mentioned at this point. When designing intervention programs in dealing with PIU, it should be kept in mind that interventions need to use different approaches based on the varying needs of men and women identified in this research. In addition, future research should continue to track whether gender differences in social anxiety continue to shrink. We advise that future research continue to investigate PIU and social anxiety as multidimensional constructs.

### Limitations

Finally, this study has a few limitations. First, both social anxiety and PIU are shown to be related to many different variables in the literature, most of which are not included in this study. This study is limited to specific relationships and gender differences. Second, the study was conducted with a nonclinical sample of college students. Therefore, results are only generalizable to similar populations. Third, this study relied on self-report measures and thus might suffer from common method bias. Other means of assessing social anxiety and PIU might reveal different results. Fourth, results show associations between the constructs under investigation, but they do not imply any causality. Thus, it would be inappropriate to conclude that social anxiety *causes* PIU or vice versa.

## Abbreviations

DSM: Diagnostic and Statistical Manual of Mental Disorders
MANOVA: multivariate analysis of variance
PIU: problematic Internet use
PIUS: Problematic Internet Use Scale
SAS: Social Anxiety Scale
